# Discovery, Development, Inventions, and Patent Trends on Mobocertinib Succinate: The First-in-Class Oral Treatment for NSCLC with EGFR Exon 20 Insertions

**DOI:** 10.3390/biomedicines9121938

**Published:** 2021-12-17

**Authors:** Mohd Imran, Shah Alam Khan, Mohammed Kanan Alshammari, Meshal Ayedh Alreshidi, Abeer Abdullah Alreshidi, Rawan Sulaiman Alghonaim, Fayez Aboud Alanazi, Sultan Alshehri, Mohammed M. Ghoneim, Faiyaz Shakeel

**Affiliations:** 1 Department of Pharmaceutical Chemistry, Faculty of Pharmacy, Northern Border University, Rafha 91911, Saudi Arabia; 2 College of Pharmacy, National University of Science and Technology, Muscat 130, Oman; shahalam@nu.edu.om; 3 Department of Pharmaceutical Care, Rafha Central Hospital, Rafha 91911, Saudi Arabia; ii_kanan101@outlook.com; 4 Department of Pharmaceutical Care, King Khaled Hospital, Hail 81411, Saudi Arabia; Meshal.alzabni@gmail.com (M.A.A.); Pailot6@hotmail.com (A.A.A.); 5 Department of Supply, Qassim Armed Forces Hospital, Qassim 51922, Saudi Arabia; Rawansgh39@gmail.com; 6 Department of Pharmaceutical Care, Al Yamamah Hospital, Riyadh 14814, Saudi Arabia; Ph.fayez90@hotmail.com; 7 Department of Pharmaceutics, College of Pharmacy, King Saud University, Riyadh 11451, Saudi Arabia; salshehri1@ksu.edu.sa; 8 Department of Pharmacy Practice, College of Pharmacy, AlMaarefa University, Ad Diriyah 13713, Saudi Arabia; mghoneim@mcst.edu.sa

**Keywords:** mobocertinib, TAK-788, EGFR mutation, NSCLC, inventions, patent

## Abstract

The majority of lung cancers are non-small-cell lung cancer (NSCLC) having a low survival rate. Recent studies have indicated the involvement of epidermal growth factor receptor (EGFR) oncogene mutations like EGFR exon 20 insertions (EGFRex20ins) mutation among NSCLC patients. The response of patients of NSCLC with the EGFRex20ins mutation to the currently available EGFR inhibitor is negligible. Mobocertinib is the first oral treatment that has been approved by the USFDA, on 15 September 2021, to treat NSCLC with the EGFRex20ins mutation. This patent review discusses the inventions and patent literature of mobocertinib that will help the scientific community to develop additional and improved inventions related to mobocertinib. The structure of mobocertinib was first reported in 2015. Therefore, this article covered the patents/patent applications related to mobocertinib from 2015 to 25 October 2021. The patent search revealed 27 patents/patent applications related to compound, method of treatment, salt, polymorph, process, composition, and drug combinations of mobocertinib. The authors foresee an exciting prospect for developing a treatment for NSCLC with EGFRex20ins mutation, and other cancers employing a combination of mobocertinib with other approved anticancer agents. The inventions related to novel dosage forms, processes, and intermediates used in the synthesis of mobocertinib are also anticipated.

## 1. Introduction

Lung cancer is the second most common form of cancer among different kinds of cancer (11.4%) and is one of the major causes of mortality worldwide [1]. The usual risk factors [2], symptoms [3], screening tests [4], and the diagnostic tests [5] are well documented for this disease. Small-cell lung cancer (SCLC) and non-small-cell lung cancer (NSCLC) are two major types of lung cancers. The NSCLC is the most frequent type of lung cancer, accounting for about 85% of all lung cancers, whereas SCLC accounts for about 15% of all lung cancers [6,7]. The low survival rate for SCLC (about 6%) and NSCLC (about 24%) is because these diseases are not detected at early stages. The treatment of NSCLC includes surgery, radiofrequency ablation, radiation therapy, chemotherapy, immunotherapy, palliative care, and targeted drug therapy [6].

Epidermal growth factor receptor (EGFR) is a member of the type 1 tyrosine kinase family. It plays a significant role in the growth, survival, and differentiation of cells. The overexpression of EGFR is implicated in the pathogenesis of human cancers, including NSCLC [8]. Many EGFR inhibitors are in clinical practice to treat NSCLC, for example, erlotinib [9], gefitinib [10], afatinib [11,12], dacomitinib [13], and osimertinib [14]. However, many types of mutations, including the EGFR exon 20 insertions (EGFRex20ins) mutation, have been reported in EGFR [15]. These mutations cause conformational changes in EGFR, reduce access of the EGFR inhibitors to their binding pockets, and lead to the development of therapeutic resistance [16,17]. The EGFRex20ins mutations are found in about 12% of NSCLC patients and have approximately 50 variants [18,19,20]. The NSCLC patients with EGFRex20ins mutations have a brief survival time as compared with NSCLC patients with other mutations because such cases are insensitive to EGFR inhibitors. Therefore, the NSCLC cases with EGFRex20ins mutations are considered an unmet medical need [20]. Amivantamab (Rybrevant), a monoclonal antibody, is one of the options to treat NSCLC patients with EGFRex20ins mutations. However, this injectable drug is not equally efficacious in patients suffering from this disease [21]. Recently, USFDA has approved mobocertinib (Exkivity) as the first oral small molecule to treat NSCLC patients with EGFRex20ins mutations [22,23]. Pre-clinical and some clinical studies of mobocertinib have concisely been reviewed [20]. Herein, we provided a comprehensive review of the inventions and patents related to mobocertinib. This review will be useful to the scientists of the pharmaceutical industry/academia working on the development of mobocertinib inventions.

## 2. Mobocertinib

**Figure 1:**
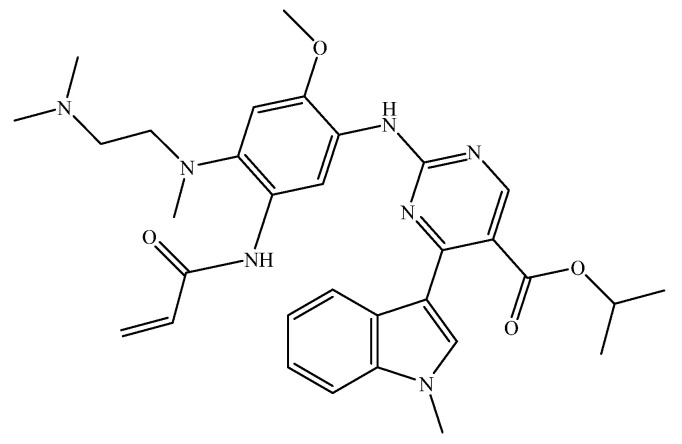
Chemical structure of mobocertinib.

Mobocertinib (Other names: AP-32788, TAK 788, TAK-788, TAK788; CAS registry number: 1847461-43-1; Molecular Formula: C_32_H_39_N_7_O_4_; Molecular weight: 585.70), chemically, is 5-Pyrimidinecarboxylic acid, 2-[[4-[[2-(dimethylamino)ethyl]methylamino]-2-methoxy-5-[(1-oxo-2-propen-1-yl)amino]phenyl]amino]-4-(1-methyl-1H-indol-3-yl)-,1-methylethyl ester (Figure 1) [24,25,26,27]. The chemical structure of this non-chiral molecule was first disclosed in 2015 [28]. The trade name of mobocertinib is Exkivity^®^, which is marketed as a capsule dosage form and contains mobocertinib succinate (CAS registry number: 2389149-74-8; Molecular Formula: C_32_H_39_N_7_O_4_.C_4_H_6_O_4_; Molecular weight: 703.8) as an active ingredient [25,29,30]. This immediate-release capsule of mobocertinib for oral administration contains 40 mg mobocertinib (free base, equivalent to 48.06 mg mobocertinib succinate) without excipients. The mobocertinib monosuccinate salt has high solubility across the physiologic pH range of pH 1.0 to pH 6.8. The highest proposed commercial dose (160 mg) of mobocertinib is soluble in 250 mL of aqueous media over the pH range of 1 to 6.8 and demonstrates a high permeability. Therefore, it exhibits the characteristics of a BCS Class 1 compound at the intended clinical doses [31].

Mobocertinib, discovered by Ariad Pharmaceuticals and developed by Takeda Pharmaceuticals, is an irreversible tyrosine kinase inhibitor (TKI) that forms a covalent bond with cysteine 797 in EGFR, leading to the inhibition of EGFR signaling, and stops EGFR exon 20 insertion mutations. It was approved by the USFDA, on 15 September 2021, to treat NSCLC with epidermal growth factor receptor (EGFR) exon 20 exon insertion mutations [24]. This is the first approval of an oral targeted therapy for patients with EGFR exon 20 insertion mutation-positive NSCLC. The route of administration, safety profile, objective response rate (ORR), and durable responses observed with mobocertinib provide a meaningful advantage over available treatments for this disease [32].

**Scheme 1:**
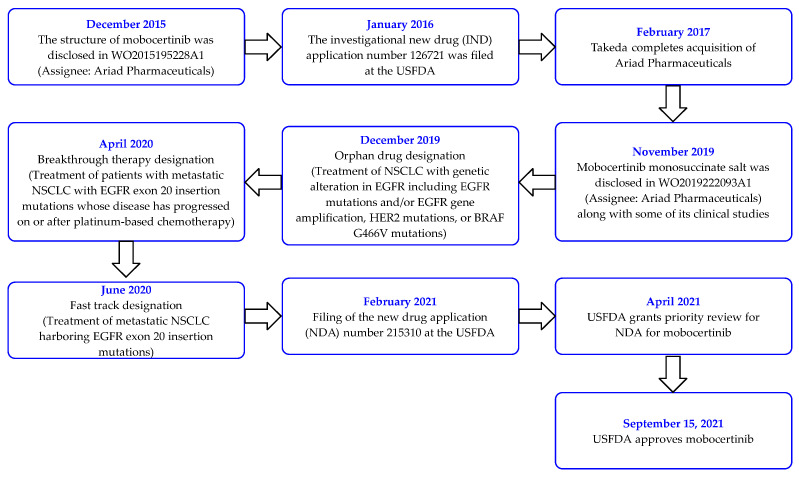
The development timeline for mobocertinib.

The mobocertinib development timeline and the summary of the Rx data/pharmacology of mobocertinib are provided in Scheme 1 and Table 1, respectively.

### Clinical Trials on Mobocertinib

The clinical trial studies on mobocertinib were searched on the clinicaltrials.gov database [36]. The search revealed eight clinical studies on mobocertinib. The summary of these clinical trials is provided in Table 2.

## 3. Patent Searching

**Scheme 2:**
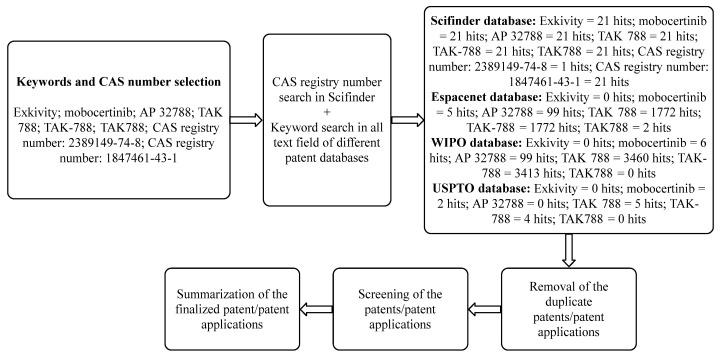
Patent searching methodology.

Searching was performed on 25 October 2021, utilizing different patent databases (Sci-finder, Espacenet, WIPO, and USPTO). The patent searching strategy is provided in Scheme 2. The patents/applications that did not cover mobocertinib (chemical structure or keywords) explicitly or implicitly in their claim section were excluded from the final list. The bibliographic data of the patents/patent applications which are cited in the text are provided in Table 3.

## 4. Patent Analysis

### 4.1. Compound Patent

**Scheme 3:**
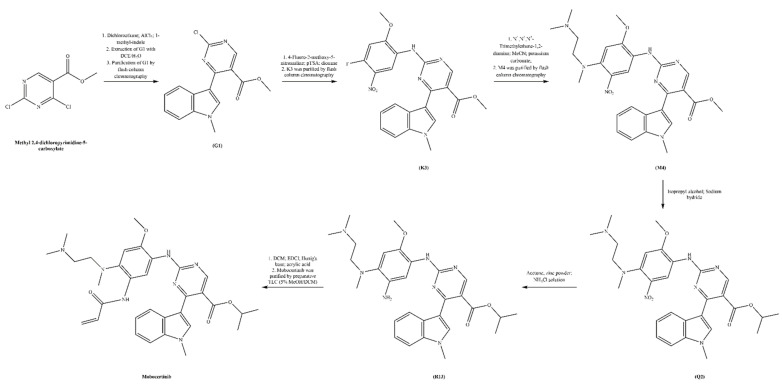
Process of preparing mobocertinib as disclosed in **US9796712B2** [37].

**US9796712B2** is related to heteroaryl compounds that selectively inhibit mutant EGFR proteins like EGFR having one or more mutations in the exon 20 domain. This patent specifically claims mobocertinib, its salts, and a pharmaceutical composition comprising them. The specification of this patent also discloses the chemical structures of the metabolites of mobocertinib (AP32960 and AP32914). Example 10 of **US9796712B2** provides the process to prepare mobocertinib (Scheme 3) [37]. Briefly, the intermediate Methyl 2-chloro-4-(1-methyl-1H-indol-3-yl)pyrimidine-5-carboxylate (**G1**) was synthesized by stirring an equimolar solution of methyl 2,4-dichloropyrimidine-5-carboxylate in dichloroethane (previously treated with aluminum chloride, 20 mmol) with 1-methyl-indole at 55 °C for 90 min. The mixture was cooled to 0 °C, methanol (5 mL) and water (10 mL) were added, and stirring was further continued at ambient temperature for 30 more min. The obtained mixture was separated into layers by the addition of water and then extracted with DCM (4 × 30 mL). The combined organic layers were dried over anhydrous magnesium sulfate, filtered, concentrated, and purified using flash chromatography to obtain pure yellow crystals of **G1**. 4.5 mmol of **G1** was heated at 100 °C for 48 h with 4-fluoro-2-methoxy-5-nitroaniline and *p*-toluenesulfonic acid in dioxane. The cooled and concentrated product upon flash chromatography yielded intermediate **K3** (methyl 2-((4-fluoro-2-methoxy-5-nitrophenyl) amino)-4-(1-methyl-1H-indol-3-yl) pyrimidine-5-carboxylate) as a brown residue. A mixture of **K3** (0.8 mmol) and a tertiary amine (*N*,*N*,*N*′-trimethylethylenediamine; 0.88 mmol) in acetonitrile in presence of K_2_CO_3_ (2.40 mmol) was stirred for 90 min at 80 °C. The resulting mixture was cooled and filtered through a pad of celite, rinsed with EtOAc, and then purified by flash chromatography to obtain **M4** (methyl 2-((4-((2-(dimethylamino) ethyl) (methyl) amino)-2-methoxy-5-nitrophenyl)amino)-4-(1-methyl-1H-indol-3-yl)pyrimidine-5-carboxylate). The mixture of **M4** (0.21 mmol) in isopropyl alcohol (3 mL) and 0.26 mmol of sodium hydride was refluxed for 5 min and then cooled to obtain a solid red compound, isopropyl 2-((4-((2-(dimethylamino)ethyl)(methyl)amino)-2-methoxy-5-nitrophenyl)amino)-4-(1-methyl-1H-indol-3-yl)pyrimidine-5-carboxylate (**Q2**). The nitrobenzene group of **Q2** was further reduced to aniline derivative by stirring its acetone solution with zinc powder (0.56 mmol) and saturated aqueous ammonium chloride (1.4 mmol) at room temperature for 30 min. The mixture was filtered through a pad of celite and concentrated to obtain isopropyl 2-((5-amino-4-((2-(dimethylamino) ethyl) (methyl) amino)-2-methoxyphenyl) amino)-4-(1-methyl-1H-indol-3-yl) pyrimidine-5-carboxylate (**R13**) as a solid product. The desired mobocertinib (isopropyl 2-((5-acrylamido-4-((2-dimethylamino) ethyl) (methyl) amino)-2-methoxyphenyl) amino)-4-(1-methyl-1H-indol-3-yl) pyrimidine-5-carboxylate) was synthesized by reacting **R13** (0.14 mmol) in DCM with 1-ethyl-3 (3-dimethylpropylamine) carbodiimide (EDCI; 0.28 mmol), Hunig’s base (0.42 mmol), and acrylic acid (0.28 mmol). The resulting mixture was concentrated in vacuo and purified by preparative TLC (5% MeOH/DCM).

**Figure 3:**
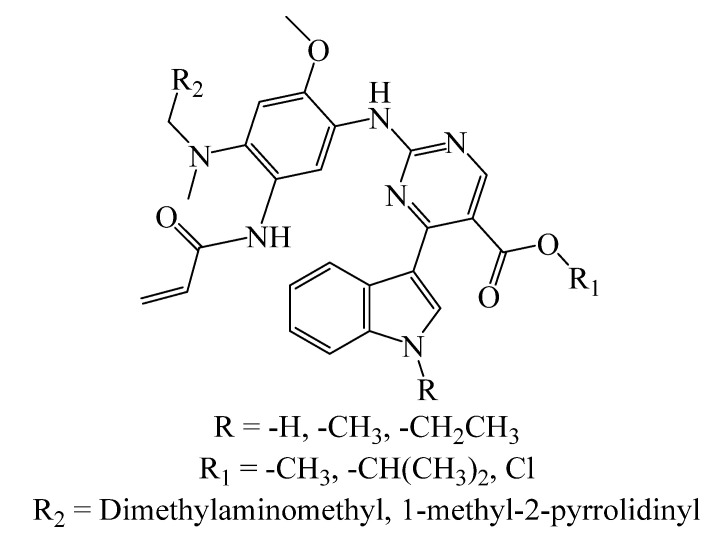
Possible basic pharmacophoric structure of mobocertinib.

The biological example records of this patent report the IC_50_ data of mobocertinib (IC_50_ ≤ 100 µM) against EGFR exon 20 ASV and NPG insertion; EGFR exon 19 deletion and T790M mutation; EGFR exon 21 L858R and T790M mutation; and Her2 exon 20 YVMA insertion employing mutated Ba/F3 and murine pro-B cell lines. The claimed compounds of **US9796712B2** [37], including mobocertinib, were declared to be effective against diseases linked with mutant EGFR activity. The compounds prepared according to examples 9, 17, 30, 34, 35, 36, 56, 66, 67, and 77 of **US9796712B2** [37] displayed equipotency (IC_50_ ≤ 100 µM) with mobocertinib. The analysis of these structures revealed the possible basic pharmacophoric structure of mobocertinib (Figure 3), wherein the indole ring can be replaced with 1H-pyrrolo[2,3-*b*]pyridine ring [37].

**US10227342B2** [38], a family member of **US9796712B2** [37], also claims mobocertinib and its pharmaceutical compositions. It also claims a method to treat NSCLC linked with one or more insertion or deletion mutations in the exon 20 domain of EGFR/HER2 using an effective amount of mobocertinib, wherein the NSCLC is resistant to other EGFR/HER2 inhibitors. **US2019218212A1** is also a family member of **US9796712B2** [37] and **US10227342B2** [38]. It claims a process for preparing mobocertinib [39].

### 4.2. Salt Patent Application

**Scheme 4:**
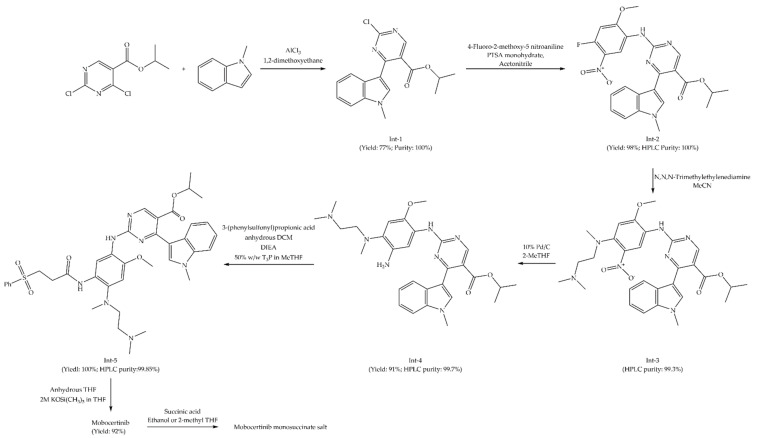
Process of preparing mobocertinib disclosed in **US2021309640A1** [40].

**US2021309640A1** discloses salts of mobocertinib (succinate, sulfate, mesylate, oxalate, hydrochloride, tosylate, hippurate, hydrobromide, and fumarate) [40]. It also discloses a process for preparing mobocertinib (Scheme 4), wherein the polymorphic Form I of mobocertinib was obtained after the recrystallization/purification of mobocertinib from ethyl acetate (EtOAc) as well as from dichloromethane (DCM). They prepared mobocertinib in six steps via five intermediates. In step 1, isopropyl 2,4-dichloropyrimidine-5-carboxylate (42.5 mmol) in DME was reacted with 1-methyl indole (44.9 mmol) in the presence of AlCl_3_ to obtain isopropyl 2-chloro-4-(1-methyl-1H-indol-3-yl)pyrimidine-5-carboxylate (intermediate 1) as a pure yellow solid in 77% yield. Intermediate 1 (258 mmol) was heated with 4-fluoro-2-methoxy-5 nitroaniline (306 mmol) and PTSA monohydrate (70 mmol) in acetonitrile under nitrogen for 19 h which, on extraction with EtOAc and usual work up, furnished isopropyl 2-((4-fluoro-2-methoxy-5-nitrophenyl) amino)-4-(1-methyl-1H-indol-3-yl) pyrimidine-5-carboxylate (intermediate 2) in 98% yield. In step 3, the 4-fluoro substituent on phenyl ring was replaced with dimethylamino) ethyl (methyl) amino group. Reaction of one equivalent of intermediate 2 and two equivalents of *N*,*N*,*N*-trimethylethylenediamine in acetonitrile produced dimethylamino)ethyl(methyl)amino)-2-methoxy-5-nitrophenyl)amino)-4-(1-methyl-1H-indol-3-yl)pyrimidine-5-carboxylate (int 3). The 5-nitro phenyl group of intermediate 3 was reduced to aniline derivatives by carrying out hydrogenation with 10% Pd/C. A yellow solid in 91% yield was obtained as intermediate 4 (isopropyl 2-((5-amino-4-((2-(dimethylamino) ethyl) (methyl)-amino)-2-methoxyphenyl) amino)-4-(1-methyl-1H-indol-3-yl)pyrimidine-5-carboxylate). In step five, a reaction mixture comprising of one equivalent of intermediate 4 and 3-(phenylsulfonyl) propionic acid (1.07 eq.) in anhydrous DCM was cooled to 2 °C, and stirred with one equivalent of *N*,*N*-diisopropylethylamine, or Hünig’s base, to yield intermediate 5 (isopropyl 2-((4-((2-(dimethylamino) ethyl)(methyl) amino)-2-methoxy-5-(3-(phenylsulfonyl) prop anamido) phenyl)amino)-4-(1-methyl-1H-indol-3-yl)pyrimidine-5-carboxylate). The intermediate 5 in tetrahydrofuran (THF) was then reacted slowly with KOSi(CH_3_)_3_ in THF to obtain mobocertinib in 92% yield. It also states that polymorphic Form I of mobocertinib (free base compound) can also be obtained using acetone, acetonitrile, chloroform, dimethylformamide (DMF), dimethylsulfoxide (DMSO), EtOAc, isobutyl acetate, methanol, 2-methoxyethanol, 2-MeTHF, dioxane, or methyl isobutyl ketone. **US2021309640A1** specifically claims succinate salt of mobocertinib along with its crystalline forms (Form-I and Form-III), pharmaceutical compositions (capsule), and a method of treating NSCLC linked with mutant EGFR/HER2 using mobocertinib succinate [40]. The patent application reports the preparation of mobocertinib succinate by the reaction of mobocertinib with succinic acid in ethanol as well as 2-methyl THF. The preparation of Form-III is reported employing a mixture of water-saturated ethyl acetate and methanol. Example 12 (phase 1/2 results), example 13 (phase 1/2 study/dose-escalation study), example 14 (expansion and extension phases), and example 15 (clinical pharmacology and pharmacokinetics) of **US2021309640A1** discuss the clinical studies on the capsule formulation of the polymorphic Form-I of mobocertinib succinate (DSC melting point: 177.5 °C; Hygroscopicity: 1.3%; Chemical stability: stable, as no change in the XRPD was observed at ambient light, 40 °C + 75% relative humidity, and at 80 °C; Water solubility: 1.9 mg/mL) on patients with advanced NSCLC refractory to standard therapy having EGFR exon 20 insertions [40]. The pharmacological data are also available in the approved label of mobocertinib and are summarized in Table 2.

### 4.3. Polymorph Patent Application

**Scheme 5:**
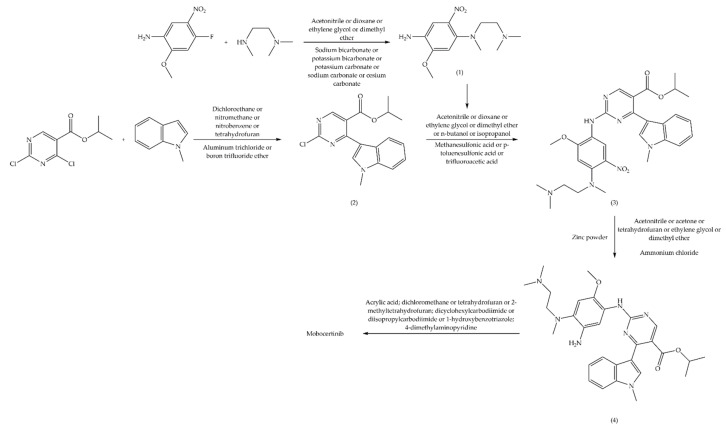
Process of preparing mobocertinib disclosed in **CN110776495A** [27].

**CN110776495A** claims crystalline form A and crystalline form B of mobocertinib having a defined X-ray powder diffraction (XRPD) pattern. It also claims the use of these crystalline forms to prepare medicaments to treat cancer [27]. Example 2 of **CN110776495A** provides a process for preparing crystalline form A of mobocertinib, wherein mobocertinib (2 g) was dissolved in 120 mL of isopropanol at 80 °C. The mixture was slowly cooled at room temperature, stirred for 5–15 h, filtered, and dried at 50 °C [27]. Example 3 of **CN110776495A** mentions the preparation of form B, wherein mobocertinib (2 g) was dissolved in 50 mL of acetone at reflux temperature. The mixture was cooled to below 10 °C, stirred, and the crystals were filtered. The crystal forms A and B of mobocertinib were found to be stable and did not show any change in their XRPD after storing at 40 °C and 75% relative humidity for one week [27]. The **CN110776495A** also provides a new process for preparing mobocertinib (Scheme 5) but does not claim it. Overall, the synthesis of mobocertinib was achieved in five steps; 2-(dimethylamino) ethyl)-5-methoxy-*N*^1^-methyl-2-nitrobenzene-1, 4-diamine (Compound 1) was prepared by reacting 4-fluoro-2-methoxy-5-nitrobenzene and *N*,*N*,*N*’-trimethylethylenediamine in an organic solvent in presence of a base such as sodium or potassium carbonate. Isopropyl 2-chloro-4-(1-methyl-1H-indol-3-yl) pyrimidine-5-carboxylate (compound 2) was synthesized separately in a Lewis acid-catalyzed reaction by condensing isopropyl 2, 4-dichloropyrimidine-5-carboxylate with *N*-methylindole in an organic solvent. Compounds 1 and 2 were reacted together in presence of an acid to form the isopropyl 2-((4(2 (dimethylamino) ethyl) (methyl) amino)-2-methoxy-5-nitrophenyl) amino-4-(1-methyl-1H-indol-3-yl) pyrimidine-5-carboxylate (compound 3) which was further reduced with zinc dust in acidic medium (ammonium chloride) to yield Isopropyl 2-(5-amino-4-((2-(dimethylamino) ethyl) (methyl) amino)-2-methoxyphenyl) amino-4-(1-methyl-1H-indol-3-yl) pyrimidine-5-carboxylate (compound 4). Finally, the desired compound, mobocertinib, was obtained by condensing compound 4 with acrylic acid in presence of condensing agents like Dicyclohexylcarbodiimide (DCC), Diisopropylcarbodiimide (DIC), or EDCI.

### 4.4. Drug Combinations with Anlotinib

Anlotinib is a clinically used receptor tyrosine kinase (RTK) inhibitor to treat NSCLC [41]. Many patent applications have been identified that claim a combination of anlotinib and other anticancer agents (mobocertinib, erlotinib, afatinib, crizotinib, ceritinib, gefitinib, dacomitinib, etc.) for the treatment of NSCLC with positive gene mutation (**CN111643503A**) [42], giant cell tumor of bone (**CN111840289A**) [43], interstitial lung disease (**CN111956649A**) [44], ovarian cancer (**CN111973747A**) [45], colorectal cancer (**CN112043702A**) [46], esophageal cancer (**CN112121048A**) [47], glioblastoma (**CN112294814A**) [48], Ewing sarcoma (**WO2020211860A1**) [49], soft tissue sarcoma (**WO2020228656A1**) [50], and SCLC (**WO2020233602A1**) [51]. The beneficial effects of the claimed inventions include enhanced curative effect and reduced dose/side effects of chemotherapeutics. However, none of these patent applications exemplify the effect of the combination of anlotinib and mobocertinib against any cancer type.

### 4.5. Other Combinations

**US2019091229A1** states a method for treating cancer (NSCLC, acute myeloid leukemia, and chronic lymphocytic leukemia) having one or more activating mutations (EGFR or HER2 exon 20 insertion mutation) using a pharmaceutical composition comprising MPC-0767 (an Hsp90 inhibitor) or its salt, alone or in combination with a protein kinase inhibitor (erlotinib, afatinib, lapatinib, dacomitinib, gefitinib, mobocertinib, poziotinib, osimertinib, and EGF816) [52].

**US2021196719A1** claims a method for preventing or treating EGFR mutation tumor diseases like NSCLC using a combination of a CDK4/6 inhibitor (abemaciclib, ribociclib, palbociclib, alvocidib, trilaciclib, voruciclib, gossypin, taurosporine, etc.) and an EGFR inhibitor (mobocertinib, osimertinib, gefitinib, erlotinib, olmutinib, dacomitinib, etc.). The combination is claimed to exhibit good effects in treating NSCLC [53].

**WO2020052575A1** states the use of a JAK kinase inhibitor (tofacitinib, itacitinib, peficitinib, baricitinib, ruxolitinib, etc.) combined with an EGFR inhibitor (mobocertinib, osimertinib, gefitinib, erlotinib, brigatinib, dacomitinib, afatinib, neratinib, lapatinib, etc.) in the manufacture of a medicament for preventing or treating an EGFR-mutated tumor disease-like NSCLC (squamous cell carcinoma and non-squamous cell carcinoma, preferably non-phosphorous cell carcinoma). The combination is stated to possess a synergistic effect [54].

**WO2020177678A1** reveals the use of a multi-target tyrosine kinase inhibitor (sunitinib, axitinib, sorafenib, nintedanib, cabozantinib, lenvatinib, regorafenib, ponatinib, pazopanib, etc.) combined with an EGFR inhibitor (mobocertinib, osiertinib, gefitinib, erlotinib, brigatinib, dacomitinib, afatinib, neratinib, lapatinib, etc.) in the preparation of a medicine for treating EGFR mutation tumor-like NSCLC (squamous cell carcinoma and non-squamous cell carcinoma). The combination is said to produce an improved therapeutic effect [55].

It is important to note that these patent applications do not exemplify the effect of MPC-0767 [52], CDK4/6 inhibitor [53], JAK kinase inhibitor [54], and multi-target tyrosine kinase inhibitor [55] in combination with mobocertinib against any cancer type.

### 4.6. Miscellaneous

**CN111760024A** claims a penetration-enhancing gold nanocluster drug-carrying targeting preparation. The preparation comprises a gold nanocluster, a cross-linking agent, and a tumor treatment drug (mobocertinib, doxorubicin, erlotinib, lapatinib, dacomitinib, gefitinib, etc.) for treating malignant tumors of breast cancer, melanoma, and prostate cancer. The claimed preparation not only promotes penetration, but also improves the targeted treatment effect on tumors and reduces the toxic and side effects of a single treatment, and has a high medical diagnosis and treatment application value [56].

**WO2019178267A2** provides a composition comprising an isolated transcription factor (Lymphoid enhancer factor 1 or Lef-1) and an EGFR inhibitor (mobocertinib) to treat degenerative lung diseases and/or conditions such as asthma, COPD, cystic fibrosis, and other forms of airway epithelial damage. The composition covers a combination of a large number of active agents along with Lef-1 [57].

**WO2021163064A2** reveals antibodies and fusion proteins that bind CCR8 for treatment of cancer alone or in a combination of other anticancer agents like mobocertinib. The application is silent about the experimental details of such a combination [58].

**WO2021183474A1** provides a method for treating a patient suffering from chronic inflammatory injury, metaplasia, dysplasia, or cancer of gastrointestinal tissue, whose method comprises administering to the patient an agent like HSP90 inhibitor or an EGFR inhibitor (mobocertinib) or a combination thereof that selectively kills or inhibits the proliferation or differentiation of pathogenic epithelial stem cells (PESCs) in the gastrointestinal tissue relative to normal epithelial stem cells in GI tissue in which the PESC is found. This application does not exemplify any data related to mobocertinib [59].

Our search revealed some patent applications claiming novel compounds as anticancer agents, including deoxynyboquinone analogs (**WO2019113345A1**) [60], pyrimidine derivatives (**WO2021000912A1**) [61], pyrazine derivatives (**WO2021033153A1**) [62], and pyrimidinones (**WO2021149817A1**) [63]. These patent applications also state the use of the claimed compounds in combination with mobocertinib for the treatment of cancer without providing any experimental evidence.

## 5. Conclusions

Mobocertinib is a promising first-in-class oral treatment for an unmet medical need (NSCLC with EGFRex20ins mutations). It is expected to increase the survival rate of the patients suffering from this disease. The patents/patent applications related to compound, process, salt, polymorphs, and combination of mobocertinib have been filed by many applicants, especially in USA and China. It would be interesting to observe how many patented mobocertinib combinations with other anticancer agents will enter the clinical trial. Many more patent applications related to novel dosage forms, new drug combinations, processes, and intermediates used in the synthesis of mobocertinib are expected to come into the public domain in the future.

## 6. Expert Opinion

A decrease in the mortality of NSCLC patients has been observed in recent times with the arrival of targeted therapy [64]. Oncogenes, for example, EGFR genes, have been identified in NSCLC patients. Therefore, the NSCLC is treated based on the oncogene mutations [65,66,67]. Many EGFR inhibitors are available in the market to treat NSCLC with common mutations [9,10,11,12,13,14,68]. The EGFRex20ins mutation is considered an uncommon mutation and has also been acknowledged as a separate subclass of EGFR mutations [69]. The NSCLC patients with EGFRex20ins mutations receive negligible benefit from the existing therapeutic option [68], which makes it an unmet medical need. In May 2021, USFDA approved the first injectable treatment (amivantamab) for NSCLC with EGFRex20ins mutations [21]. However, mobocertinib is a recently approved first-in-class oral innovative treatment of NSCLC with EGFRex20ins mutations.

**Figure 4:**
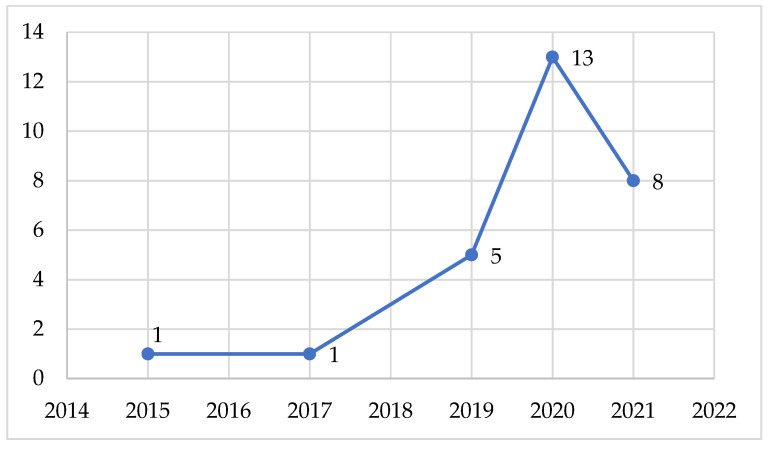
Publication year of the patents/patent applications cited in the text.

It is imperative to understand the prior inventions related to therapy to develop its improved inventions/innovations. Accordingly, the patent literature search of mobocertinib was undertaken (Scheme 2). The patent search revealed 27 patents/patent applications belonging to 25 patent families (Table 3). The first Patent Cooperation Treaty (PCT) patent application claiming mobocertinib was published in 2015 [28], wherein the compound patent of mobocertinib was granted in 2017 in the USA [37]. The method of use patent of mobocertinib was granted in 2019 in the USA [38]. The process patent application of the compound patent family has been abandoned in the USA [39]. However, a continuity application number 17/463,457 has been filed at the USPTO in 2021, which may be published soon. Most of the patent applications on mobocertinib were published in 2020 (Figure 4).

**Figure 5:**
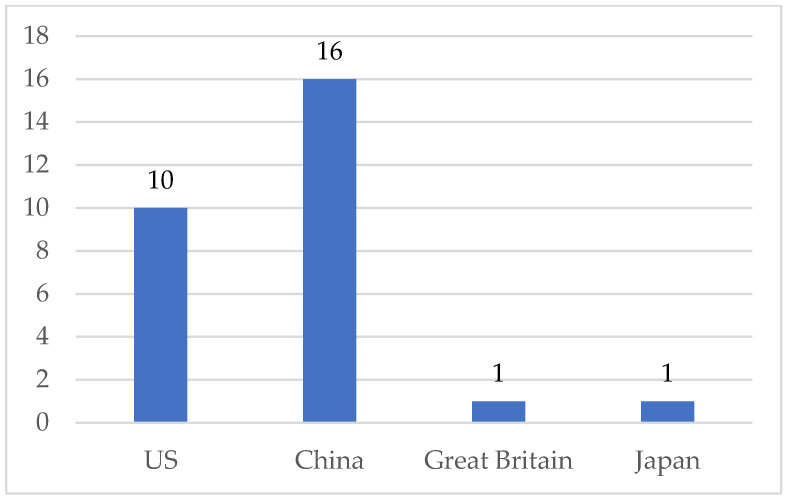
The country of the priority application filing of the patents/patent applications cited in the text.

The maximum number of priority patent applications have been filed in China followed by the USA (Figure 5). One patent application each has been filed in Japan and Great Britain.

**Figure 6:**
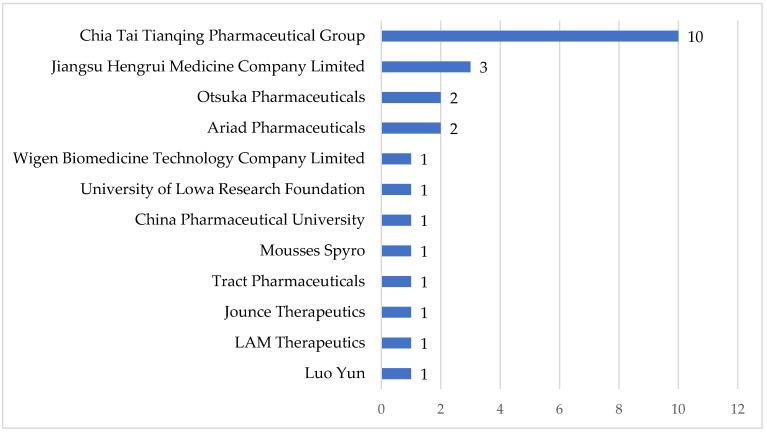
The number of patent families filed by different applicants.

The 25 patent families cited in the text were filed by different applicants (Figure 6). It is important to note that only two patent families (compound patent family and the mobocertinib succinate patent family) have been filed by Ariad Pharmaceuticals, who discovered and developed mobocertinib [37,38,39,40]. Most of the patent applications have been filed by Chia Tai Tianqing Pharmaceutical Group, which are related to combinations of anlotinib with other anticancer agents like mobocertinib (Table 3, Figure 6).

**Figure 7:**
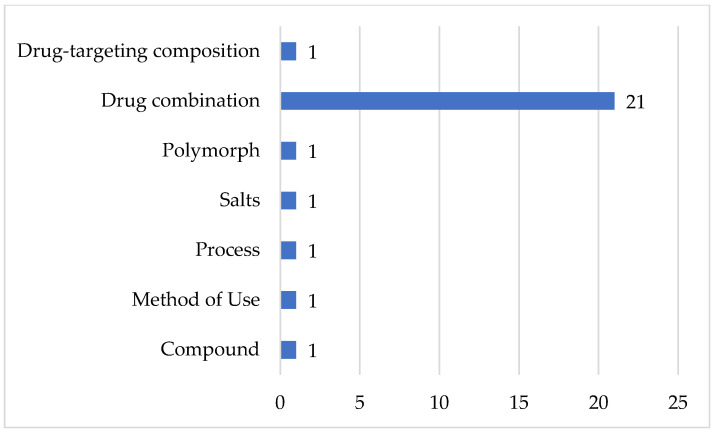
The types of patents filed for mobocertinib.

Different types of patents are possible for a pharmaceutical compound including patents claiming compound, salt, isomer, polymorph, process, intermediate used in the process, drug impurity, method of treatment, different dosage forms, particle size, and drug combinations [70,71,72,73]. The types of patents/patent applications related to mobocertinib are mentioned in Figure 7.

The compound patent and the method of use patent of mobocertinib expire on 13 May 2035, and were filed by Ariad Pharmaceuticals (Table 3) [37,38]. In early 2017, Takeda acquired Ariad Pharmaceuticals and became the owner of the patents filed by Ariad Pharmaceuticals. These two patents are eligible for patent term extension up to five years [70,71,72,73]. If this happens, these patents will expire by 2040. Our analysis of the structure-activity relationship resulted in the possible basic pharmacophoric structure of mobocertinib (Figure 3). We trust that medicinal chemists can use the isostere and bioisotere concepts to develop more potent and safer drugs like mobocertinib. The mobocertinib succinate salt patent application was published in 2019 and is under examination at the time of writing this review [40]. If this patent application is granted, then the estimated expiry of the granted patent will be 13 May 2039. The authors trust that the mobocertinib succinate salt patent will be listed in the orange book of the USFDA [74,75]. It will also be eligible for a 5-year patent term extension and may become the governing patent for the generic drug launch of mobocertinib in the USA market. The authors also see an opportunity for a salt switch strategy [76] to develop other salts of mobocertinib with improved pharmaceutical properties. The compound patent family [37,38,39] also has published patent applications related to the process for preparing mobocertinib [39] (Scheme 3). The salt patent application [40] and polymorph patent application [27] also provide other processes for preparing mobocertinib (Scheme 4 and Scheme 5). The process patents of drugs help to reduce the price of the marketed dosage form [70]. Accordingly, we anticipate more patent filing related to the process for preparing mobocertinib from the drug suppliers. Anticancer therapy generally involves the use of a combination of drugs. Accordingly, many patent applications related to the combination of mobocertinib with other anticancer agents have been identified [42,43,44,45,46,47,48,49,50,51,52,53,54,55]. However, most of these patent applications are silent about the clinical data of the combination of mobocertinib with other drugs. Therefore, clinical studies for the combination of mobocertinib with other anticancer agents are recommended.

The patent literature revealed important inventions related to mobocertinib. However, there remains a great scope for developing inventions related to pharmaceutical compositions (different dosage forms, combinations, particle size, process intermediates, etc.). The combination of mobocertinib with pemetrexed and carboplatin is in a clinical trial for NSCLC (Table 1). There are many agents in clinical trials to treat NSCLC with EGFRex20ins mutations [15,18]. It will be worth assessing the combinations of these drugs with mobocertinib to treat this unmet need. The authors believe that there exists an exciting prospect for developing a treatment for NSCLC with EGFRex20ins mutations, giant cell tumor of the bone [43], interstitial lung disease [44], ovarian cancer [45], colorectal cancer [46], oesophageal cancer [47], glioblastoma [48], Ewing sarcoma [49], and soft tissue sarcoma [50] employing a combination of mobocertinib with other approved anticancer agents.

## Figures and Tables

**Figure 2:**
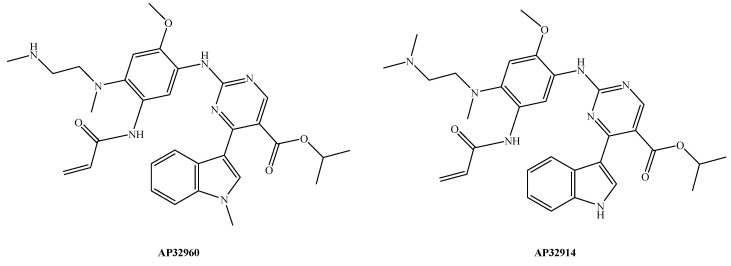
Chemical structures of *N*-demethylated active metabolites of mobocertinib (AP32960 and AP32914).

**Table 1:** R_x_ data of mobocertinib.

Parameter	Summary
Drug data	Proprietary name: Exkivity; Active ingredient: Mobocertinib succinate; Dosage Form: Capsule; Route: Oral; Strength: 48.06 mg of mobocertinib succinate (Equivalent to 40 mg of mobocertinib base); Applicant: Takeda Pharmaceuticals [33]
Application data	Application number: N215310; Review type: Priority review; Orphan status: Orphan drug; Approval date: 15 September 2021; Marketing Status: Prescription [33]
Exclusivity data	Exclusivity type (Expiry date): New chemical entity (September 15, 2026) [33]
Indication (Use Code)	NSCLC with EGFR exon 20 exon insertion mutations whose disease has progressed on or after platinum-based chemotherapy (U-3220) [30,33]
Recommended Dose	160 mg/day with/without food till disease progression/unacceptable toxicity. The dose can be reduced based on the adverse reactions [29,30,34,35]
Warning/Precautions	Exkivity may lead to QTc prolongation and/or torsades de pointes. If this happens, reduce the dose or discontinue Exkivity based on the severity of QTc prolongation. Discontinue Exkivity if the patient shows induction of interstitial lung disease/pneumonitis, cardiac toxicity, diarrhea, and embryo-fetal toxicity. Additionally, avoid concomitant use of CYP3A inhibitors with Exkivity that can prolong QTc [29,30]
Adverse effects	Diarrhoea, rash, nausea, stomatitis, vomiting, decreased appetite, paronychia, fatigue, dry skin, musculoskeletal pain, increased amylase/lipase/creatinine, and decreased potassium/hemoglobin/magnesium/lymphocytes [29,30,34]
Contraindication	No data is available [29,30]
Drug interaction	Avoid concomitant use of CYP3A inhibitors as well as inducers like itraconazole and rifampin [24,30]
Absorption	Tmax = 4 h; Absolute bioavailability = 37%; C_max_ 45.8 ng/mL; AUC_0-inf_ = 862 ngh/mL [34]
Volume of distribution	3509 L (38%) at steady-state [29,30]
Metabolism	Mobocertinib is metabolized by CYP3A via *N*-demethylation to produce two equipotent metabolites, namely, AP32960 (Figure 2) and AP32914 (Figure 2) [24]
Protein binding	Mobocertinib = 99.3%; AP32960 = 99.5%; AP32914 = 98.6% [25,29,30]
Route of elimination	Mobocertinib (Faeces, 76%; Urine, 4%), AP32960 (Faeces, 12%; Urine, 1%), AP32914 (Undetected) [29,30]
Half-life	Mobocertinib = 18 h; AP32960 = 24 h; AP32914 = 18 h [29,30]
Clearance	Mobocertinib = 138 L/h; AP32960 = 149 L/h; AP32914 = 159 L/h [29,30]
Toxicity	Cardiac toxicity; Ocular toxicity; embryo-fetal toxicity [29,30] Grade 3 or 4 abnormality: Decreased red blood cells (3.5%); Decreased lymphocytes (15%); Decreased platelets (0.9); Decreased leukocytes (0%); Increased creatinine (2.7%); Increased amylase (13%); Increased lipase (10%); Decreased potassium (5.3%); Increased alkaline phosphatase (1.8%); Decreased albumin (1.8%); Decreased magnesium (2.7%); Increased alanine aminotransferase (2.7%); Increased aspartate aminotransferase (1.8%); Decreased sodium (0.9%) [29,30] No data about overdose are available [29,30]

**Table 2:** Clinical trials on mobocertinib.

Title of the Study	Condition (Study Type; Phase; NCT Number; Number Enrolled)	Status (Study Start Date (SSD); Study Completion Date (SCD); Last Update Date (LUD))	Sponsor/Collaborators (Funder Type; Location)
An expanded access protocol for mobocertinib in refractory NSCLC participants with EGFR Exon20 insertion mutations	NSCLC (Expanded access; Marketed; NCT04535557; Not mentioned)	Approved for marketing (SSD: Not mentioned; SCD: Not mentioned; LUD: 27 September 2021)	Millennium Pharmaceuticals/Takeda (Industry; USA)
A study of TAK-788 in adults with NSCLC in combination with pemetrexed/carboplatin	NSCLC (Interventional; Phase 1 and 2; NCT02716116; 395)	Recruiting (SSD: 16 June 2016; SCD: 13 March 2023; LUD: 25 May 2021)	Millennium Pharmaceuticals/Takeda (Industry; USA)
A study to evaluate the impact of management strategies on gastrointestinal-related adverse events in participants with NSCLC harboring EGFR Exon20 insertion mutations receiving TAK-788	NSCLC (Interventional; Phase 2; NCT04576208; zero)	Withdrawn (SSD: 30 November 2020; SCD: 31 July 2022; LUD: 17 December 2020)	Takeda (Industry; not mentioned)
A study to evaluate drug-drug interaction of TAK-788 with itraconazole and rifampin in healthy adult participants	Healthy volunteers (Interventional; Phase 1; NCT03928327; 24)	Completed (SSD: 2 May 2019; SCD: 16 August 2019; LUD: 21 August 2020)	Millennium Pharmaceuticals/Takeda (Industry; USA)
A study to evaluate the effect of high-fat meals on TAK-788 pharmacokinetics in healthy adult participants	Healthy volunteers (Interventional; Phase 1; NCT04441255; 14)	Completed (SSD: 1 July 2020; SCD: 10 August 2020; LUD: 5 September 2021)	Takeda (Industry; USA)
A study of mobocertinib capsules in people with severe kidney problems and people with healthy kidneys	Renal impairment (Interventional; Phase 1; NCT04056455; 24)	Recruiting (SSD: 10 March 2020; SCD: 31 August 2022; LUD: 13 July 2021)	Millennium Pharmaceuticals/Takeda (Industry; USA)
A study to evaluate pharmacokinetics and safety of oral mobocertinib in participants with moderate or severe hepatic impairment and normal hepatic function	Hepatic impairment (Interventional; Phase 1; NCT04056468; 24)	Recruiting (SSD: 23 August 2019; SCD: 31 December 2021; LUD: 16 June 2021)	Millennium Pharmaceuticals/Takeda (Industry; USA)
A study to assess absolute bioavailability of mobocertinib and to characterize mass balance, pharmacokinetics, metabolism, and excretion of carbon-14 ([14C])-mobocertinib in healthy male participants	Healthy Volunteers (Interventional; Phase 1; NCT03811834; 7)	Completed (SSD: 22 January 2019; SCD: 11 March 2019; LUD: 30 July 2021)	Millennium Pharmaceuticals/Takeda (Industry; USA)

**Table 3:** Bibliography of the cited patents.

Patent/Patent Application Number (Assignee; Publication Date; Earliest Priority Date; Estimated Expiry Date)	Legal Status on 25 October 2021 (International Patent Classification)	Family Members
**US9796712B2** (Ariad Pharma; 24 October 2017; 19 June 2014; 13 May 2035)	Patented case (C07D401/14, C07D403/04, C07D405/12, C07D471/04, C07F9/6558, A61K31/506, C07D403/14)	AU2015277786B2, AU2019206024A1, BR112016029662A2, CA2949793A1, CL2016003222A1, CL2017003103A1, CN106559991B, CN110526912A, CO2017000386A2, CR20170011A, CU20160185A7, CY1121359T1, DK3157916T3, EA034691B1, ECSP17003553A, EP3157916B1, EP3409669B1, EP3778584A1, ES2715500T3, GEP20207111B, GEP20197011B, HRP20190407T1, HUE042390T2, IL248859A, JP6230205B2, JP6546630B2, KR20170016861A, LT3157916T, MA40240B1, ME03334B, MX361802B, PE20170268A1, PH12016502453A1, PL3157916T3, RS58541B1, SG10201913753VA, SG11201610517PA, SI3157916T1, TN2016000560A1, TR201903322T4, TR201903322T4, UA121657C2, WO2015195228A1, ZA201608224B
**US10227342B2** (Ariad Pharma; 12 March 2019; 19 June 2014; 13 May 2035)	Patented case (C07D401/14, C07D403/04, C07D405/12, C07D471/04, C07F9/6558, A61K31/506, C07D403/14)	
**US2019218212A1** (Ariad Pharma; 18 July 2019; 19 June 2014; 13 May 2035)	Abandoned on 28 April 2020 The child continuity data state that application number 17/463,457 has been filed on 31 August 2021, which may be published soon. (C07D401/04, C07D401/14, C07D403/04, C07D403/14, C07D405/12, C07D405/14, C07D471/04, C07F9/6558)	
**US2021309640A1** (Ariad Pharma; 7 October 2021; 14 May 2018; 13 May 2039)	Ready for Examination (A61P35/00, C07D403/04)	AU2019269372A1, BR112020023107A2, CA3099737A1, CN112313223A, EP3793993A1, JP2021523208A, KR20200144579A, TW202015687A, WO2019222093A1
**CN110776495A** (LUO YUN; 11 February 2020; 30 July 2018; 30 July 2038)	Under examination (A61K31/506, A61P35/00, C07D403/04)	None
**CN111643503A** (Chia Tai Tianqing Pharmaceutical Group; 11 September 2020; 4 March 2019; 4 March 2039)	Publication (A61K31/4709, A61K45/06, A61P35/00)	None
**CN111840289A** (Chia Tai Tianqing Pharmaceutical Group; 30 October 2020; 28 April 2019; 28 April 2039)	Publication (A61K31/4709, A61K31/675, A61K31/704, A61K31/7048, A61K33/243, A61P29/00, A61P35/00)	None
**CN111956649A** (Chia Tai Tianqing Pharmaceutical Group; 20 November 2020; 20 May 2019; 20 May 2039)	Publication (A61K31/475, A61K45/06, A61P11/00)	None
**CN111973747A** (Chia Tai Tianqing Pharmaceutical Group; 24 November 2020; 23 May 2019; 23 May 2039)	Publication (A61K31/4709, A61K45/06, A61P15/08, A61P35/00)	None
**CN112043702A** (Chia Tai Tianqing Pharmaceutical Group; 8 December 2020; 5 June 2019; 5 June 2040)	Publication (A61K31/4709, A61K45/06, A61P35/00)	None
**CN112121048A** (Chia Tai Tianqing Pharmaceutical Group; 25 December 2020; 6 June 2019; 8 June 2040)	Publication (A61K31/337, A61K31/4709, A61K31/7048, A61K33/24, A61P35/00)	None
**CN112294814A** (Chia Tai Tianqing Pharmaceutical Group; 2 February 2021; 31 July 2019; 31 July 2040)	Publication (A61K31/4188, A61K31/4709, A61K45/06, A61P35/00)	None
**WO2020211860A1** (Chia Tai Tianqing Pharmaceutical Group; 22 October 2020; 19 April 2019; 19 April 2040)	No national phase entry (A61K31/4709, A61K45/06, A61P35/00, C07D401/12)	None
**WO2020228656A1** (Chia Tai Tianqing Pharmaceutical Group; 19 November 2020; 10 May 2019; 11 May 2040)	No national phase entry (A61K31/4709, A61K31/704, A61P35/00)	None
**WO2020233602A1** (Chia Tai Tianqing Pharmaceutical Group; 26 November 2020; 20 May 2019; 20 May 2040)	No national phase entry (A61K31/4709, A61P35/00, A61P35/04)	None
**US2019091229A1** (LAM Therapeutics; 28 March 2019; 27 September 2017; 27 September 2038)	Abandoned on April 15, 2020 (A61K31/095, A61K31/337, A61K31/357, A61K31/4184, A61K31/4709, A61K31/4725, A61K31/506, A61K31/52, A61K31/5377, A61K31/553, A61K9/00, A61P35/00)	AU2018341571A1, BR112020006009A2, CA3076915A1, CN111372588A, EP3687542A1, JP2020535173A, KR20200077518A, TW201919634A, US2020253979A1, WO2019067666A1
**US2021196719A1** (Jiangsu Hengrui Medicine Company Limited; 1 July 2021; 23 May 2018; 22 May 2039)	Ready for Examination (A61K31/506, A61K31/519, A61K31/5377, A61P35/00)	CA3100867A1, CN111818925A, EP3811946A1, JP2021525244A, KR20210013155A, TW202002964A, WO2019223716A1
**WO2020052575A1** (Jiangsu Hengrui Medicine Company Limited; 19 March 2020; 12 September 2018; 11 September 2039)	National phase entry in China and Taiwan (A61K31/519, A61K31/675, A61P35/00)	CN112533606A, TW202024067A
**WO2020177678A1** (Jiangsu Hengrui Medicine Company Limited; 10 September 2020; 4 March 2019; 3 March 2040)	National phase entry in China and Taiwan (A61K31/404, A61P35/00, C07D403/04, C07D519/00)	CN113301895A, TW202100150A
**CN111760024A** (China Pharmaceutical University; 13 October 2020; 24 July 2020; 24 July 2040)	Request for examination filed (A61K31/282, A61K31/5365, A61K33/243, A61K39/395, A61K41/00, A61K47/52, A61K47/62, A61K49/04, A61K49/08, A61K49/14, A61K49/18, A61K49/22, A61P35/00, B22F9/24, B82Y30/00, B82Y40/00)	None
**WO2019178267A2** (University Of Iowa Research Foundation; 19 September 2019; 13 March 2018; 13 March 2039)	National phase entry in USA (C12N5/07, C12N5/0789)	US2021255170A1
**WO2021163064A2** (Jounce Therapeutics; 19 August 2021; 14 February 2020; 9 February 2041)	National phase entry in USA (A61P35/00, C07K16/28)	US2021277129A1
**WO2021183474A1** (Tract Pharmaceuticals; 16 September 2021; 9 March 2020; 9 March 2041)	No national phase entry (A61K39/00, A61K39/395, A61P1/00, A61P1/04, A61P19/02, A61P25/00)	None
**WO2019113345A1** (Mousses Spyro; 13 June 2019; 6 December 2017; 6 December 2038)	No national phase entry (A61K31/4745, A61P35/00)	None
**WO2021000912A1** (Wigen Biomedicine Technology Company Limited; 7 January 2021; 4 July 2019; 2 July 2040)	National phase entry in China and USA (A61K31/506, A61P35/00, A61P35/02, C07D471/06)	CN112174961A, US2021230161A1
**WO2021033153A1** (Otsuka Pharmaceuticals; 25 February 2021; 20 August 2019; 20 August 2040)	National phase entry in Taiwan (A61K31/4985, A61P35/00, C07D487/04)	TW202124383A
**WO2021149817A1** (Otsuka Pharmaceuticals; 29 July 2021; 24 January 2020; 22 January 2041)	No national phase entry (A61K31/506, A61K31/519, A61K39/395, A61K45/00, A61P35/00)	None

## Data Availability

This work did not report any data.
